# Integrated Metabolomic and Transcriptomic Analyses Reveal Phenylpropanoid–Associated Variation Among Three *Dendrobium huoshanense* Germplasm Materials

**DOI:** 10.3390/metabo16090612

**Published:** 2026-08-27

**Authors:** Peipei Wei, Binbin Du, Xingen Zhang, Guohui Li, Fang Li, Ping Zhang, Jiayi Dou, Li Zou, Junjie Yang, Yujuan Wang, Jun Dai

**Affiliations:** 1 Engineering Technology Research Center of Plant Cell Engineering, Lu’an 237012, China; 02000148@wxc.edu.cn (P.W.); 02000188@wxc.edu.cn (B.D.);; 2 College of Life and Health, West Anhui University, Lu’an 237012, China; 3 Anhui Dabieshan Huohu Technology Co., Ltd., Lu’an 237200, China

**Keywords:** *Dendrobium huoshanense*, flower, metabolomics, transcriptomics, phenylpropanoid biosynthesis, germplasm variation, candidate genes

## Abstract

**Background: ***Dendrobium huoshanense* flowers are a potentially valuable medicinal resource, but metabolic variation among different germplasm materials remains incompletely characterized. This study aimed to characterize metabolic and transcriptional differences among three *D. huoshanense* germplasm materials and to explore gene–metabolite associations related to phenylpropanoid metabolism. **Methods:** Untargeted LC–MS metabolomics and transcriptome sequencing were used to comparatively profile flowers of three *D. huoshanense* germplasm materials (DH-1, DH-2, and DH-3). **Results:** Metabolomic profiling detected 4357 metabolic features putatively assigned to 12 chemical classes and revealed clear separation among the three materials. A total of 1292, 1861, and 2035 differentially accumulated metabolic features were identified in DH-1 vs. DH-2, DH-1 vs. DH-3, and DH-2 vs. DH-3, respectively. Transcriptome analysis identified 33,665 expressed genes, including 3365, 5244, and 5964 DEGs in the respective pairwise comparisons. Phenylpropanoid biosynthesis was recurrently enriched across all three DEG comparisons. Exploratory gene–metabolite analysis identified associations involving phenylpropanoid–related candidate genes, including *PAL*, *C4H*, *4CL/4CL–like*, *HCT*–*like*, *CSE*–*like*, *COMT/OMT*–*like*, and *CAD*, with the DH-2 vs. DH-3 comparison showing the most extensive molecular differences. **Conclusions:** The three *D. huoshanense* germplasm materials exhibited distinct metabolic and transcriptional profiles, providing candidate genes and metabolic features for subsequent targeted validation.

## 1. Introduction

*Dendrobium huoshanense* C.Z. Tang et S.J. Cheng is a perennial epiphytic herb of the family Orchidaceae that is endemic to the Dabie Mountain region, particularly Huoshan County in Anhui Province, China [1,2]. Owing to its long history of medicinal use and outstanding therapeutic value, this species has long been regarded as an authentic and superior source of *Dendrobii Officinalis Caulis* in traditional Chinese medicine and has historically been honored as the “first of the nine immortal herbs” [3].

The stems of *D. huoshanense* are the principal medicinal organ and have been reported to exhibit a wide range of pharmacological activities, including immunomodulatory, anti–inflammatory, hypoglycemic, and other health–promoting effects [4,5,6]. These bioactivities are mainly attributed to its diverse bioactive constituents, such as polysaccharides, bibenzyls, flavonoids, alkaloids, and other phenolic compounds [7,8,9,10]. In particular, polysaccharides from *D. huoshanense* have been shown to possess significant immunoregulatory activity [10], whereas bibenzyl compounds isolated from the stems display marked anti–inflammatory potential [11]. In addition, transcriptomic and metabolite–based studies have highlighted the importance of stem tissues in the biosynthesis and accumulation of medicinally relevant compounds in this species [12,13,14].

Although the stems have long been the primary focus of research and utilization, increasing attention has recently been directed toward other organs of *Dendrobium*, especially the flowers, because these tissues also contain abundant bioactive compounds and may represent valuable yet underutilized medicinal resources [15,16]. Studies on flowers of *D. officinale* and related medicinal *Dendrobium* species have demonstrated the presence of diverse flavonoids, amino acids, fatty acids, phenolic substances, and other metabolites with antioxidant and nutritional potential [15]. Moreover, recent studies have shown that the functional components of *Dendrobium* are dynamically regulated across developmental stages and tissues, further highlighting the importance of non–stem organs in resource development [15]. At the molecular level, integrated transcriptomic and metabolomic analyses of *D. officinale* flowers have revealed pronounced developmental changes in both metabolite accumulation and gene expression, indicating that floral tissues are subject to complex metabolic regulation [17,18]. In contrast, research on *D. huoshanense* flowers remains limited and is still largely restricted to the identification of a few constituents or preliminary biological evaluations [19]. A systematic and in–depth characterization of the metabolite composition of *D. huoshanense* flowers, together with the molecular basis underlying metabolite accumulation, is still lacking.

Cultivar selection is a fundamental aspect of medicinal plant cultivation because it directly influences agronomic traits, environmental adaptability, biomass accumulation, and, most importantly, the abundance and composition of bioactive metabolites that determine medicinal quality and efficacy. In *Dendrobium*, substantial differences in metabolite accumulation and related gene expression have been documented among tissues, developmental stages, growth years, and species [13,17,20,21]. For example, tissue– and organ–specific transcriptomic studies in *D. officinale* have identified marked spatial variation in flavonoid–and polysaccharide–related pathways [22,23], while integrated metabolomic and transcriptomic analyses have demonstrated stage–dependent and year–dependent changes in major medicinal metabolites [17,21]. Comparative studies across *Dendrobium* species have likewise revealed pronounced divergence in flavonoid biosynthesis and regulatory networks [20,24]. These findings strongly suggest that different germplasm materials of *D. huoshanense* may also differ in morphology, physiology, and phytochemical composition. Elucidating such variation, particularly at the metabolic and transcriptional levels, is therefore essential for germplasm identification, quality control, and the breeding of elite materials with enhanced medicinal value. Nevertheless, comparative studies focusing specifically on the flowers of different *D. huoshanense* germplasm materials remain scarce.

In the present study, integrated metabolomic and transcriptomic analyses were performed to characterize molecular variation in the flowers of three *D. huoshanense* germplasm materials (DH-1, DH-2, and DH-3). Specifically, the objectives were to (1) systematically profile and compare metabolic features among the three materials and identify differentially accumulated features and (2) characterize transcriptional differences and explore their associations with metabolite variation and candidate metabolic pathways. This study provides a comparative molecular dataset for understanding metabolic diversity in *D. huoshanense* flowers and for identifying candidate genes and metabolic features for subsequent validation.

## 2. Materials and Methods

### 2.1. Plant Materials, Cultivation Conditions, and Sampling

**Figure 1:**
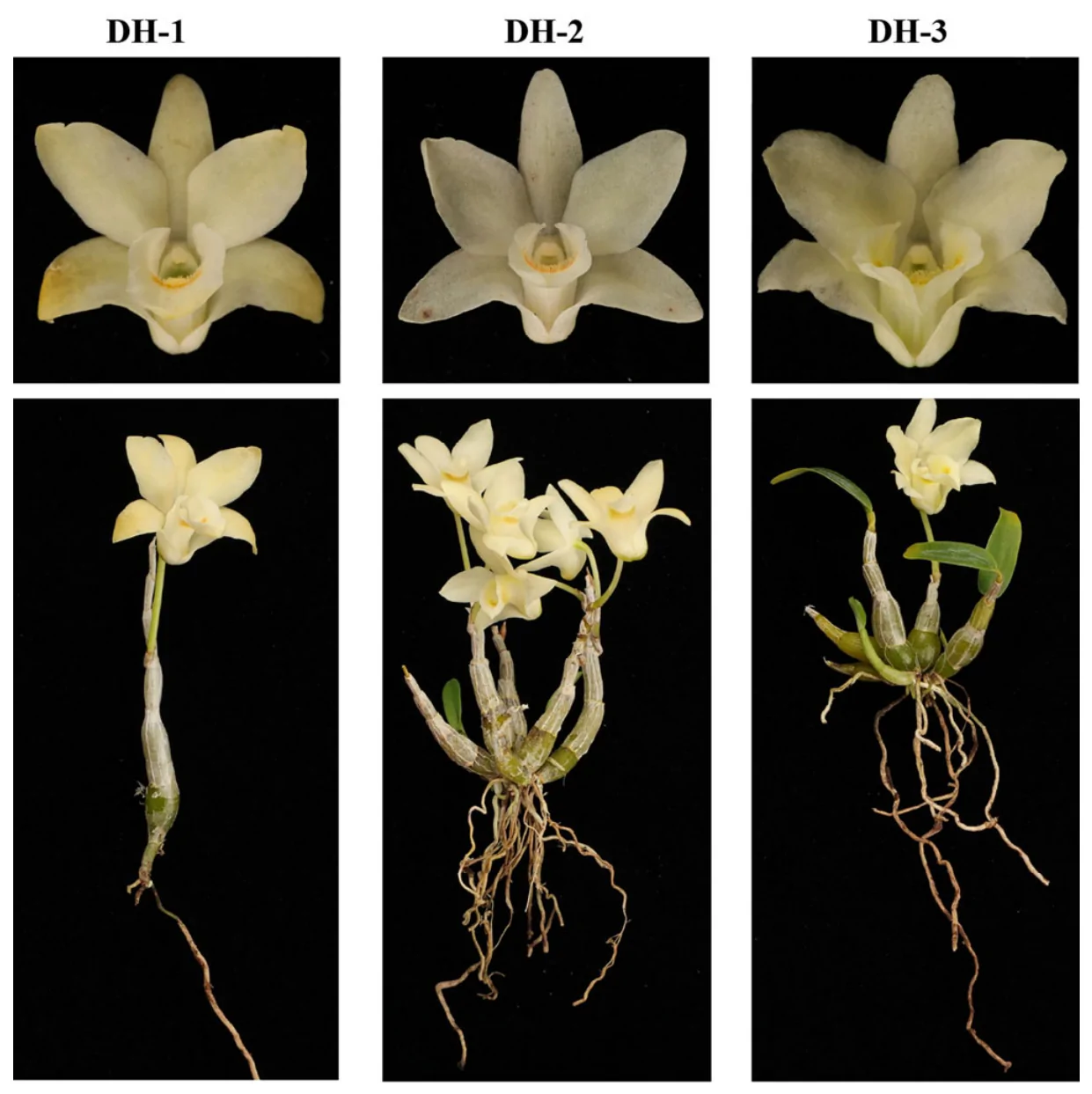
Representative flowers and whole plants of the three *D. huoshanense* germplasm materials used in this study: DH-1, DH-2, and DH-3.

Three *Dendrobium huoshanense* germplasm materials were used in this study: DH-1 (Huohu No. 1; germplasm accession HS–SH–001), DH-2 (Huohu No. 2; HS–SH–002), and DH-3 (Huohu No. 3; HS–SH–003) (Figure 1). These materials are maintained in the Dabieshan Medicinal Plant Germplasm Resource Bank of West Anhui University (Lu’an, China). The three materials originated from the same wild population and underwent the same propagation and cultivation procedures. Plants were initially propagated by sterile sowing of seeds obtained from wild capsules, followed by approximately 12 months of in vitro culture and 12 months of seedling acclimatization. The acclimatized plants were subsequently transferred to the pine–forest cultivation site and grown for three years under the same management regime before sampling. Thus, all experimental plants were mature flowering plants with the same field–cultivation age and comparable propagation histories.

The plants were cultivated at a *D. huoshanense* cultivation base in Taipingfan, Huoshan County, Anhui Province, China, under a pine–forest understory cultivation system. The forest canopy closure was approximately 0.6–0.7. A 10–15 cm–thick stone–based substrate layer was established on the forest floor, and plants were grown at approximately 20 cm × 20 cm spacing. Air temperature and relative humidity followed the natural mountain microclimate and were not artificially regulated. During dry periods, plants were irrigated by atomized spraying for approximately 15 min in the early morning, whereas artificial irrigation was suspended during rainy periods. Each plant clump received approximately 15 g of decomposed rapeseed–cake organic fertilizer as top dressing in March each year. No chemical fertilizers or pesticides were applied during cultivation. All three materials were maintained at the same cultivation site under the same general cultivation and management regime.

Flower samples were collected between 09:00 and 10:00 on 7 May 2025. Sampling was based on a predefined morphological developmental stage rather than simply on the population–level peak flowering period. Only fully open, nonsenescent flowers with fully expanded sepals and petals and without visible wilting, browning, or mechanical damage were collected for all three materials. For each material, six biological replicates were prepared, with each replicate comprising entire flowers pooled from eight individual plants. Accordingly, the pooled floral sample, rather than an individual plant, constituted the experimental unit. Samples were immediately flash–frozen in liquid nitrogen after collection and stored at −80 °C until metabolomic and transcriptomic analyses.

### 2.2. Metabolomics Analysis

Flower samples from the three *D. huoshanense* germplasm materials (DH-1, DH-2, and DH-3) were subjected to untargeted LC–MS analysis by Novogene Co., Ltd. (Beijing, China). For metabolite extraction, 100 mg of liquid–nitrogen–ground flower tissue was mixed with 500 μL of 80% aqueous methanol. The samples were vortexed, incubated on ice for 5 min, and centrifuged at 15,000× *g* for 20 min at 4 °C. An aliquot of the supernatant was diluted with MS–grade water to a final methanol concentration of 53% and centrifuged again at 15,000× *g* for 20 min at 4 °C. The resulting supernatant was collected for LC–MS analysis. Pooled quality–control (QC) samples were prepared by mixing equal volumes of all experimental samples. Blank samples containing 53% aqueous methanol were processed using the same procedure.

Chromatographic separation was performed using a Vanquish UHPLC system (Thermo Fisher Scientific, Germering, Germany) coupled to a Q Exactive HF mass spectrometer. An ACQUITY UPLC BEH Amide column was maintained at 40 °C with a flow rate of 0.2 mL min^−1^. In positive–ion mode, mobile phase A consisted of 95% acetonitrile containing 0.1% formic acid and 10 mM ammonium acetate, and mobile phase B consisted of 50% acetonitrile containing 0.1% formic acid and 10 mM ammonium acetate. In negative–ion mode, mobile phases A and B consisted of 95% and 50% acetonitrile, respectively, each containing 10 mM ammonium acetate. The gradient was programmed as follows: 0–1.5 min, 98% A; 1.5–7 min, 98–0% A; 7–9 min, 0% A; 9–9.1 min, 0–98% A; and 9.1–12 min, 98% A. Mass spectra were acquired in positive– and negative–ionization modes over an *m*/*z* range of 100–1500. The electrospray–ionization parameters were as follows: spray voltage, 3.5 kV; sheath–gas flow rate, 35 psi; auxiliary–gas flow rate, 10 L min^−1^; capillary temperature, 320 °C; S–lens RF level, 60; and auxiliary–gas heater temperature, 350 °C. MS/MS spectra were acquired using data–dependent scanning.

Raw data files were converted to mzXML format using ProteoWizard (v3.0.11781). Peak detection and quantification were performed using XCMS (v4.0), and features were aligned across samples according to retention time and *m*/*z*. Feature annotation was performed by matching accurate masses, adduct information, and MS/MS spectra against a high–quality spectral database using a mass tolerance of 10 ppm. Signals detected in blank samples were removed. Peak areas were normalized as follows: raw peak area/[total peak area of the sample/total peak area of QC1]. Features with coefficients of variation greater than 30% in the QC samples were excluded. A 30% QC–CV threshold was adopted as a reproducibility criterion for this exploratory untargeted metabolomics dataset to exclude analytically unstable features while avoiding excessive loss of potentially informative signals [25]. KEGG, HMDB, and LIPID MAPS were used for feature annotation.

Multivariate statistical analyses, including principal component analysis (PCA) and hierarchical clustering analysis (HCA), were performed in R. Orthogonal partial least squares–discriminant analysis (OPLS–DA) was conducted using the MetaboAnalystR (v4.0) package to identify metabolites contributing to discrimination among sample groups. Variable importance in projection (VIP) scores were calculated from the OPLS–DA model. Differentially accumulated metabolites (DAMs) were defined based on |log_2_(fold change)| ≥ 1 and FDR < 0.05, while OPLS–DA–derived VIP values > 1 were used as complementary evidence for prioritizing differential metabolites.

### 2.3. Transcriptome Sequencing and Analysis

Total RNA was extracted from flower samples using a CTAB–based method combined with PBIOZOL reagent (Beijing Bomais Technology Development Co., Ltd., Beijing, China) and dissolved in DEPC–treated water. RNA concentration was measured using a Qubit fluorometer (Thermo Fisher Scientific, Germering, Germany), and RNA integrity was assessed using a Qsep400 Bio–Fragment Analyzer based on the RNA Quality Number (RQN). Six biological replicates were analyzed for each of the three *D. huoshanense* germplasm materials, resulting in 18 RNA–seq libraries. Poly(A)–containing mRNA was enriched from total RNA using poly–T oligo–attached magnetic beads (Thermo Fisher Scientific, Oslo, Norway) and fragmented. First–strand cDNA was synthesized using random hexamer primers, followed by strand–specific second–strand synthesis using dUTP. After end repair, A–tailing, adapter ligation, size selection, amplification, and purification, the libraries were quantified using Qubit and real–time PCR, and their size distributions were assessed using a Bioanalyzer. Libraries with insert sizes of approximately 250–350 bp were subjected to paired–end Illumina sequencing.

Raw FASTQ reads were processed using fastp (v0.23.2) to remove adapter–containing reads, reads containing poly–N sequences, and low–quality reads. Q20, Q30, and GC content were calculated from the resulting clean reads. Clean reads were aligned to the *D. huoshanense* reference genome (version *D. huoshanense*.v20200604.chromosome) using HISAT2 (v2.2.1) with the corresponding gene–model annotation file. Gene–level raw integer counts were generated using featureCounts (v2.0.6). FPKM values were calculated separately for descriptive assessment and visualization of gene–expression abundance. Differential expression analysis was performed using DESeq2 (v1.42.0) with the raw integer count matrix generated by featureCounts as input; FPKM values were not used for differential–expression testing. DESeq2 performed count normalization through its internal size–factor estimation and modeled gene counts using a negative binomial distribution. Pairwise comparisons were performed among DH-1, DH-2, and DH-3. The resulting *p*–values were adjusted using the Benjamini–Hochberg procedure, and genes with an adjusted *p*–value ≤ 0.05 and |log_2_(fold change)| ≥ 1 were considered differentially expressed genes (DEGs). Replicate–level principal component analysis and hierarchical clustering were performed using transformed count data to assess biological–replicate consistency and potential technical effects. GO and KEGG pathway enrichment analyses of DEGs were performed using the clusterProfiler R package (v4.8.1).

### 2.4. Exploratory Gene–Metabolite Correlation Analysis

DEGs mapped to the KEGG phenylpropanoid biosynthesis pathway were selected for gene–metabolite correlation analysis. Differentially accumulated metabolic features annotated within the phenylpropanoids and polyketides class were included as the corresponding metabolomic dataset. For each pairwise comparison, Pearson correlation coefficients were calculated between the normalized expression levels of the selected DEGs and the normalized abundances of the selected metabolic features. Gene–metabolite pairs with an absolute Pearson correlation coefficient (|*R*|) > 0.90 were retained as strong associations and used for network visualization. The resulting networks were constructed to characterize associations between phenylpropanoid–related gene–expression variation and metabolic–feature abundance.

## 3. Results

### 3.1. Metabolite Profiling of Three D. huoshanense Germplasm Materials

**Figure 2:**
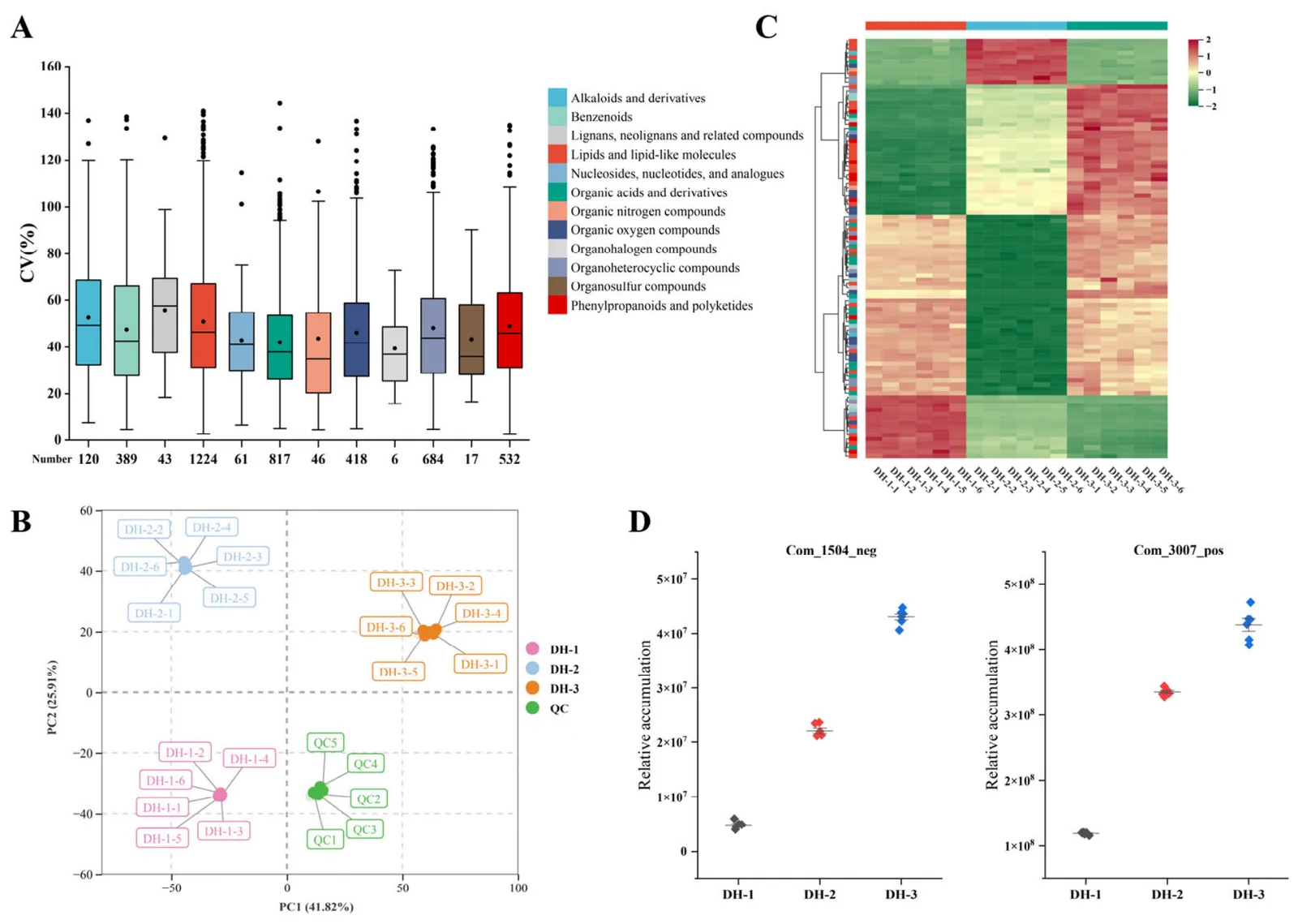
Comparative metabolic–feature profiling of three *D. huoshanense* germplasm materials. (**A**) Distribution of coefficients of variation (CV%) across putatively assigned chemical classes; numbers below the boxes indicate the number of features in each class. (**B**) PCA score plot based on the total metabolic–feature profiles of three *D. huoshanense* germplasm materials. (**C**) Heatmap of the 100 features with the largest PCA contributions; columns are biological replicates and rows are features, scaled as indicated by the color key. (**D**) Relative signal abundance of Com_1504_neg and Com_3007_pos, individual points represent biological replicates, and horizontal bars and error bars indicate mean ± SE.

Untargeted UHPLC–HRMS analysis generated a final processed dataset containing 4357 metabolic ion features across the positive–and negative–ionization datasets. These features were retained after blank–based background filtering, relative–peak–area normalization, and exclusion of features with coefficients of variation greater than 30% in the pooled QC samples. Putative database and spectral annotations assigned the retained features to 12 chemical classes (Figure 2A and Table S1). Because authentic standards were not analyzed and complete compound–level deconvolution of isotope peaks, adducts, in–source fragments, and signals detected across ionization modes was not demonstrated, the 4357 entries are reported as metabolic features rather than as 4357 unique, structurally confirmed metabolites. The largest putatively annotated feature categories were lipids and lipid–like molecules (1224 features), organic acids and derivatives (817 features), organoheterocyclic compounds (684 features), and phenylpropanoids and polyketides (532 features).

PCA clearly distinguished the metabolic–feature profiles of the three materials. The first two principal components, PC1 and PC2, explained 41.82% and 25.91% of the total variance, respectively, and clearly separated the three materials, indicating substantial metabolic divergence among DH-1, DH-2, and DH-3 (Figure 2B). Further analysis of the contributions of individual metabolites to PCA showed that, among the top 100 contributing features, lipids and lipid–like molecules (29), organic acids and derivatives (16), and organoheterocyclic compounds (14) represented the largest categories (Figure 2C and Table S2). Among the features with high contributions to the PCA separation, Com_1504_neg and Com_3007_pos showed higher relative peak areas in DH-3 than in the other two plant materials (Figure 2D).

### 3.2. Analysis of Differentially Accumulated Metabolites Among Germplasm Materials

**Figure 3:**
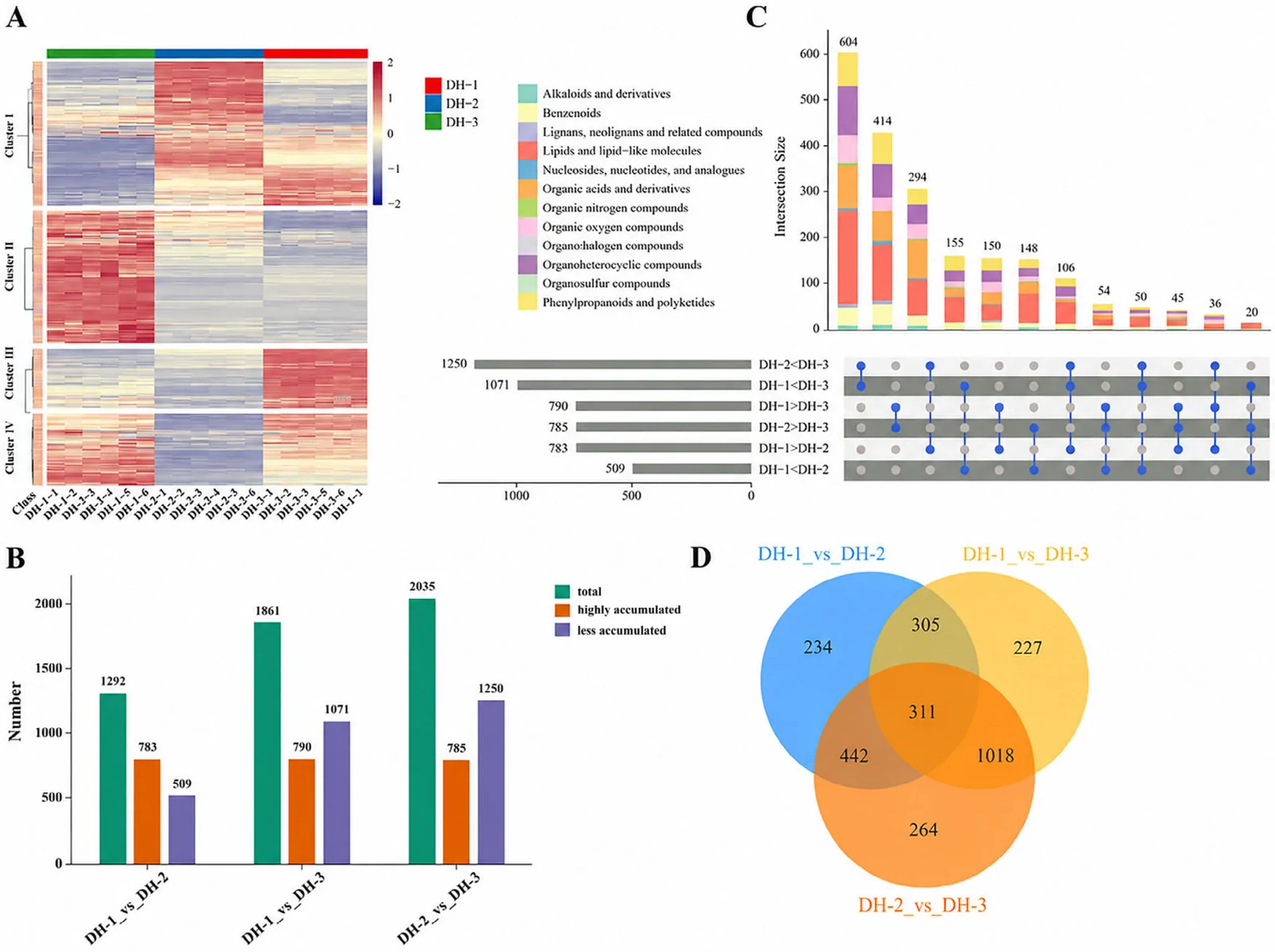
Analysis of differentially accumulated metabolites (DAMs) among the three *D. huoshanense* germplasm materials. (**A**) HCA heatmap clustering of metabolites into four clusters (I–IV) based on accumulation patterns. (**B**) Numbers of total, more highly accumulated, and less accumulated candidate features in each pairwise comparison. (**C**) UpSet plot of directional comparison categories. Each row specifies a direction (for example, DH-2 < DH-3 means lower relative accumulation in DH-2 than in DH-3); connected blue dots define an intersection, gray dots indicate directional categories that are not included in a given intersection, vertical bars give the number of features in that intersection, horizontal bars give the total size of each directional category, and stacked colors denote putative chemical classes. (**D**) Venn diagram showing the overlap of shared and unique DAMs among the three groups.

To further evaluate metabolic differences among the three *D. huoshanense* germplasm materials, hierarchical cluster analysis (HCA) was performed, and the metabolic features were broadly grouped into four clusters (Figure 3A). Most metabolites in Cluster I accumulated preferentially in DH-2, whereas Cluster II contained a greater number of metabolites that accumulated in DH-3 than in the other two materials. Cluster III was characterized by metabolites predominantly enriched in DH-1, whereas Cluster IV included metabolites that were relatively abundant in DH-3 but less abundant in DH-2 (Figure 3A).

In addition, variable importance in projection (VIP) values were calculated for each metabolite in the orthogonal partial least squares–discriminant analysis (OPLS–DA) for the three pairwise comparisons: DH-1 vs. DH-2, DH-1 vs. DH-3, and DH-2 vs. DH-3. The corresponding OPLS–DA models showed R^2^X(cum)/R^2^Y(cum)/Q^2^(cum) values of 0.733/1.000/0.998, 0.782/1.000/0.998, and 0.802/1.000/0.999, respectively (Table S3). Permutation tests further supported the robustness of the three models, with the original Q^2^ values exceeding those of the permuted models (Figure S1). DAMs were defined using |log_2_FC| > 1 and FDR < 0.05, whereas OPLS–DA–derived VIP > 1 were used as complementary evidence for prioritizing differential features. A large number of DAMs were detected in the three comparison groups, including 1292 DAMs in DH-1 vs. DH-2 (783 more highly accumulated and 509 less accumulated in DH-1), 1861 in DH-1 vs. DH-3 (790 more highly accumulated and 1071 less accumulated in DH-1), and 2035 in DH-2 vs. DH-3 (785 more highly accumulated and 1250 less accumulated in DH-2) (Figure 3B and Table S4).

Comparative analysis of the three DAM sets identified 311 shared metabolites. Meanwhile, DH-1 vs. DH-2, DH-1 vs. DH-3, and DH-2 vs. DH-3 contained 234, 227, and 264 specific DAMs, respectively (Figure 3D and Table S5). Further examination of DAM accumulation patterns revealed clear differences among the three materials. UpSet analysis indicated that DH-3 had 604 features specifically more highly accumulated and 414 specifically less accumulated relative to the other materials, whereas the corresponding counts were 150 and 155 for DH-1 and 148 and 294 for DH-2 (Figure 3C and Table S6).

### 3.3. Transcriptome Analysis and Identification of Differentially Expressed Genes

To characterize transcriptional variation associated with the observed metabolic differences among the three *D. huoshanense* germplasm materials, transcriptome sequencing was performed. After filtering and quality control, a total of 128.30 Gb of clean data was obtained, with an average Q30 value of 96.84% and an average GC content of 45.23% (Table S7). All samples were successfully mapped to the *D. huoshanense* reference genome, resulting in the identification of 33,665 expressed genes.

Consistent with the DAM analysis, differentially expressed genes (DEGs) were identified from the three pairwise comparisons, DH-1 vs. DH-2, DH-1 vs. DH-3, and DH-2 vs. DH-3, using the thresholds of |log_2_(fold change)| ≥ 1 and adjusted *p*–value (padj) < 0.05. In total, 8165 DEGs were identified, including 3365 DEGs in DH-1 vs. DH-2 (1851 upregulated and 1514 downregulated), 5244 in DH-1 vs. DH-3 (1761 upregulated and 3483 downregulated), and 5964 in DH-2 vs. DH-3 (1934 upregulated and 4030 downregulated) (Figure S2 and Table S8).

Functional enrichment analysis of the DEGs was then performed to clarify their potential biological roles. Gene Ontology (GO) enrichment analysis of the top 30 enriched terms showed that molecular function was the predominant category in all three comparison groups (Figure S3 and Table S9). Seventeen GO terms were shared among the three comparisons, including two biological process terms, “DNA integration” (GO:0015074) and “DNA metabolic process” (GO:0006259), and 15 molecular function terms: “terpene synthase activity” (GO:0010333), “carbon–oxygen lyase activity, acting on phosphates” (GO:0016838), “carbon–oxygen lyase activity” (GO:0016835), “lyase activity” (GO:0016829), “magnesium ion binding” (GO:0000287), “ADP binding” (GO:0043531), “RNA–DNA hybrid ribonuclease activity” (GO:0004523), “endoribonuclease activity” (GO:0004521), “heme binding” (GO:0020037), “tetrapyrrole binding” (GO:0046906), “oxidoreductase activity, acting on paired donors, with incorporation or reduction of molecular oxygen” (GO:0016705), “endonuclease activity” (GO:0004519), “iron ion binding” (GO:0005506), “hydrolase activity, hydrolyzing O–glycosyl compounds” (GO:0004553), and “hydrolase activity, acting on glycosyl bonds” (GO:0016798) (Figure S3 and Table S9).

**Figure 4:**
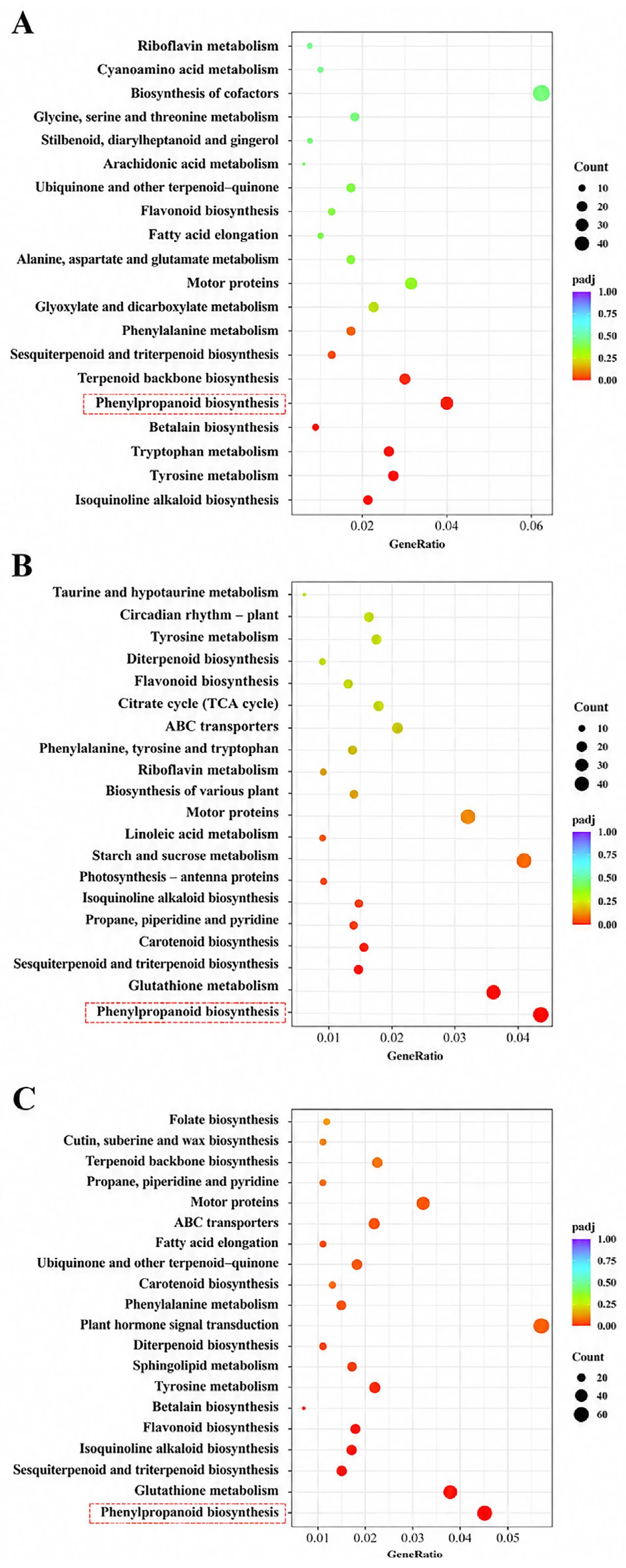
KEGG pathway enrichment of DEGs in (**A**) DH-1 vs. DH-2, (**B**) DH-1 vs. DH-3, and (**C**) DH-2 vs. DH-3. The *x*–axis shows GeneRatio, defined as the number of DEGs assigned to a pathway divided by the number of DEGs included in the enrichment analysis. Bubble size indicates DEG count and bubble color indicates the Benjamini–Hochberg–adjusted *p*–value (padj). Red dashed boxes highlight the phenylpropanoid biosynthesis pathway.

KEGG pathway enrichment analysis was further conducted to investigate the biological functions and key pathways associated with the DEGs. The results are shown in Figure 4. In the DH-1 vs. DH-2 comparison, DEGs were mainly enriched in phenylpropanoid biosynthesis, terpenoid backbone biosynthesis, and tyrosine metabolism (Figure 4A). In DH-1 vs. DH-3, DEGs were primarily enriched in phenylpropanoid biosynthesis, starch and sucrose metabolism, and glutathione metabolism (Figure 4B). In the DH-2 vs. DH-3 comparison, DEGs were mainly enriched in phenylpropanoid biosynthesis, glutathione metabolism, and plant hormone signal transduction (Figure 4C). Notably, DEGs from all three comparisons were consistently enriched in the phenylpropanoid biosynthesis pathway.

### 3.4. Exploratory Phenylpropanoid–Focused Gene–Metabolite Correlation Analysis

Because phenylpropanoid biosynthesis was recurrently enriched among the DEG comparisons, we further examined this pathway using an exploratory gene–metabolite correlation approach. DEGs assigned to the KEGG phenylpropanoid biosynthesis pathway were correlated with differentially accumulated metabolic features annotated within the broad phenylpropanoids and polyketides class, using |Pearson’s r| > 0.90 to identify strong associations. Given that this chemical class comprises metabolites from multiple biosynthetic branches, the resulting correlations were interpreted as exploratory associations.

The three comparison groups showed distinct patterns of candidate gene composition (Table S10). In DH-1 vs. DH-2, most highly correlated genes were associated with aromatic amino acid metabolism and related secondary metabolic processes, rather than canonical phenylpropanoid biosynthetic steps, such as tyrosine decarboxylase, phenylacetaldehyde synthase, and primary amine oxidase, suggesting a relatively limited contribution of the core phenylpropanoid pathway in this comparison. In contrast, the DH-1 vs. DH-3 comparison identified several typical phenylpropanoid biosynthetic genes, including *phenylalanine ammonia–lyase* (*PAL*), *4–coumarate–CoA ligase* (*4CL/4CL–like*), *shikimate O–hydroxycinnamoyltransferase* (*HCT–like*), *caffeoylshikimate esterase* (*CSE*), and *caffeic acid 3–O–methyltransferase* (*COMT*), together with several peroxidase genes, showing associations between phenylpropanoid–related gene–expression variation and metabolic–feature abundance. A similar but more extensive pattern was observed in the DH-2 vs. DH-3 comparison, in which *PAL*, *cinnamate 4–hydroxylase* (*C4H*), *4CL/4CL–like*, *shikimate O–hydroxycinnamoyltransferase* (*HCT–like*), *CSE*, *caffeoyl–CoA O–methyltransferase* (*CCoAOMT*), and *cinnamyl alcohol dehydrogenase* (*CAD*) were identified, showing more extensive differences in the expression of phenylpropanoid–associated candidate genes (Table S10).

**Figure 5:**
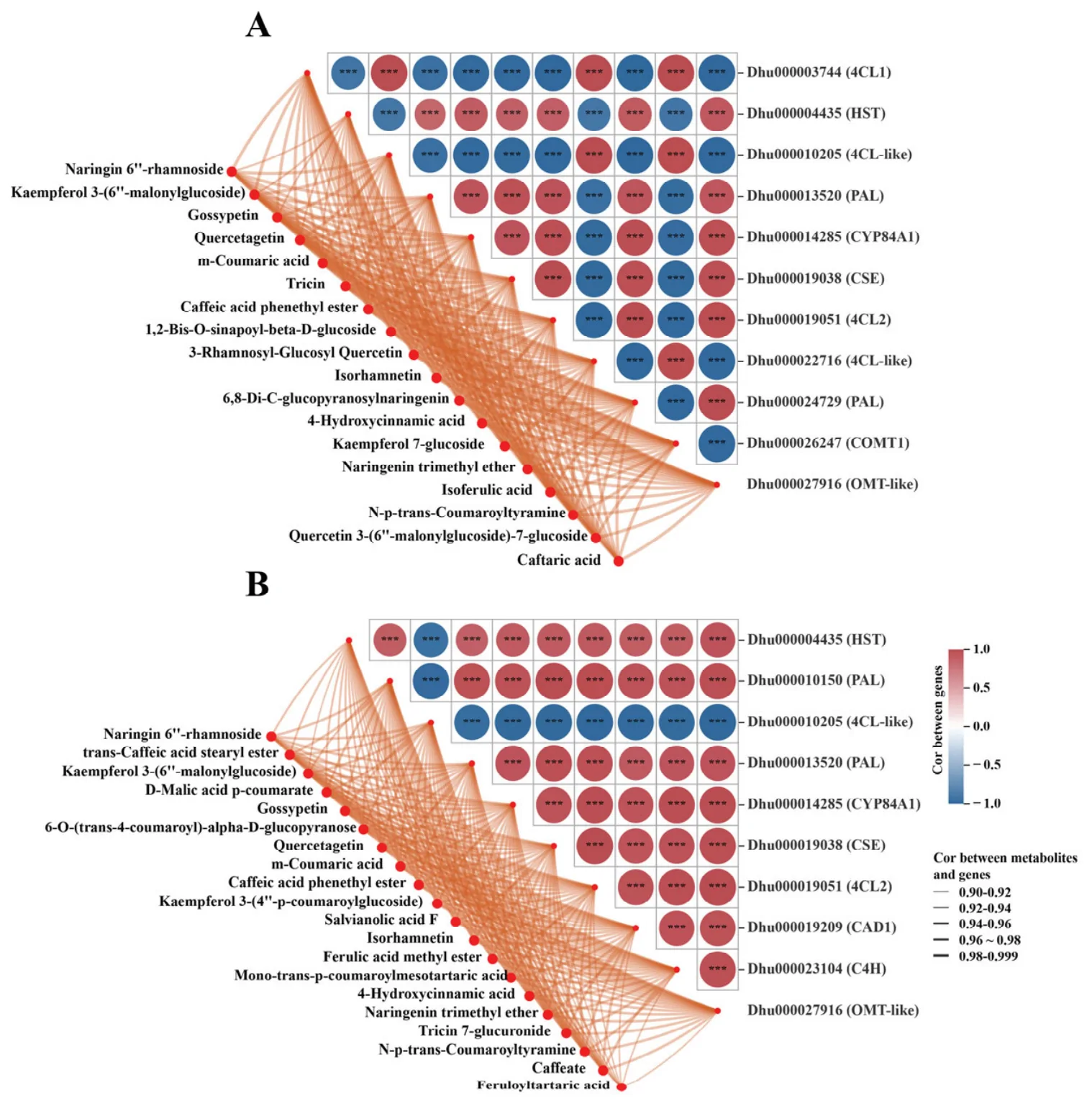
Exploratory Pearson correlation networks between putatively annotated metabolic features and candidate genes in (**A**) DH-1 vs. DH-3 and (**B**) DH-2 vs. DH-3. Red dots represent metabolic features, and solid lines represent feature–gene correlations with |*R*| > 0.90, and edge thickness is proportional to |*R*|. Red and blue in the gene–gene correlation matrices indicate positive and negative correlations, respectively. Asterisks denote two–sided significance tests (*** *p* < 0.001).

To further clarify the relationships between candidate genes and representative metabolites, correlation networks were constructed for the DH-1 vs. DH-3 and DH-2 vs. DH-3 comparisons (Figure 5). In both networks, several structural genes, including *HCT–like*, *PAL*, *4CL/4CL–like*, *CSE*, *C4H*, *COMT/OMT–like*, and *CAD*, were connected with multiple DAMs annotated as phenylpropanoids and polyketides, such as m–coumaric acid, 4–hydroxycinnamic acid, caffeate, caftaric acid, ferulic acid methyl ester, and related phenolic derivatives, indicating close associations between transcript variation and metabolite accumulation. Notably, the DH-2 vs. DH-3 comparison showed a denser exploratory correlation network than DH-1 vs. DH-3, indicating more extensive associations between the selected candidate genes and metabolic features in this comparison (Figure 5).

**Figure 6:**
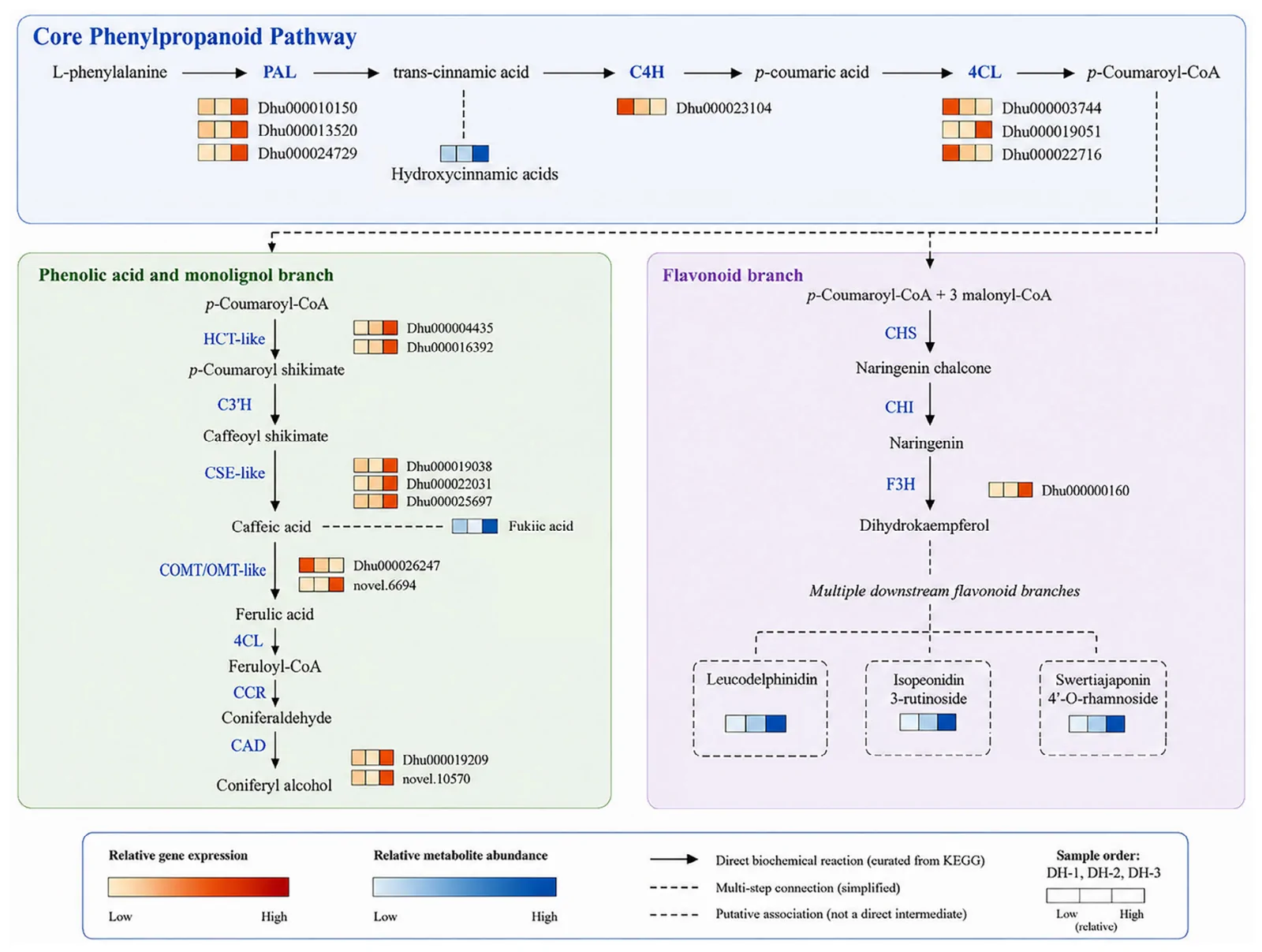
Simplified phenylpropanoid–related pathway in *D. huoshanense* flowers. Candidate genes and representative differentially accumulated metabolic features are mapped onto the core phenylpropanoid pathway and its associated phenolic acid/monolignol and flavonoid branches. Solid arrows indicate curated direct biochemical reactions, dashed arrows indicate simplified multi–step pathway connections, and dotted lines indicate putative associations rather than direct biochemical reactions. Heatmaps show the relative gene–expression levels and metabolic–feature abundances in DH-1, DH-2, and DH-3.

To summarize the biosynthetic context of these candidate genes and metabolites, a simplified phenylpropanoid pathway was constructed based on the KEGG pathway map and the integrated omics results (Figure 6). This pathway diagram highlighted the core route from phenylalanine to hydroxycinnamate intermediates and further showed the phenolic acid/monolignol and flavonoid–related branches. Several candidate genes, including *PAL*, *C4H*, *4CL*, *HCT–like*, *CSE*, *COMT*, and *CAD*, were positioned at key enzymatic steps, while representative differential metabolites were mapped to the corresponding downstream branches (Figure 6). Together, the pathway schematic summarizes the exploratory associations observed between phenylpropanoid–related candidate genes and selected metabolic features.

## 4. Discussion

The present study revealed marked differences in untargeted metabolic features and gene–expression profiles among the three *D. huoshanense* germplasm materials. Phenylpropanoid biosynthesis was consistently enriched in the DEG analyses across all three pairwise comparisons and was therefore selected for further exploratory integration with the metabolomic data. This recurrent enrichment suggests that phenylpropanoid–related processes may be associated with, and potentially contribute to, the observed molecular differences among the three materials. Phenylpropanoid metabolism is a central plant metabolic network that produces hydroxycinnamates, flavonoids, lignins, coumarins, and phenolic acids, which participate in plant growth, pigmentation, defense, and responses to environmental stimuli [26,27].

Among the three pairwise comparisons, DH-2 vs. DH-3 showed the largest numbers of both DAMs and DEGs and involved a broader set of phenylpropanoid–associated candidate genes, including *PAL*, *C4H*, *4CL/4CL–like*, *HCT–like*, *CSE–like*, *COMT/OMT–like*, and *CAD.* This pattern indicates more extensive molecular differences in phenylpropanoid–associated genes and metabolic features in the DH-2 vs. DH-3 comparison, rather than demonstrating greater pathway remodeling or regulatory activity. In contrast, DH-1 vs. DH-2 was dominated by genes associated with aromatic amino acid metabolism and related secondary metabolic processes, whereas DH-1 vs. DH-3 showed an intermediate pattern. Thus, the three pairwise comparisons differed in the extent and composition of the observed molecular variation, with comparisons involving DH-3 generally showing more pronounced differences.

The candidate genes identified here are consistent with the established biochemical framework of phenylpropanoid metabolism [28,29]. *PAL*, *C4H*, and *4CL* mediate pathway entry and precursor activation [30], whereas *HCT–like* acyltransferases and *CSE* participate in shikimate– and caffeoyl–related conversions that shape downstream metabolic flux [31,32]. *COMT–like O–methyltransferases* and *CAD* further support divergence in downstream phenolic conversion [33]. The correlation networks and pathway summary diagram provide additional support for this interpretation by showing that these structural genes were associated with multiple representative hydroxycinnamate– and phenolic–related metabolites, particularly in the DH-2 vs. DH-3 comparison. Thus, the floral metabolic differences observed among materials are more plausibly explained by coordinated variation at multiple enzymatic steps than by isolated terminal–branch changes [34,35].

Previous studies in Dendrobium have demonstrated developmental variation in floral metabolite accumulation and gene expression, indicating that flower metabolism is sensitive to developmental context [36]. The present study extends these observations by documenting material–associated metabolic and transcriptional differences in D. huoshanense flowers. Previous investigations of medicinal Dendrobium have focused predominantly on stem–derived constituents and their biological properties [37,38], whereas the present dataset provides complementary molecular information for floral tissues. Nevertheless, the current untargeted and correlation–based data alone are insufficient to establish the medicinal value or quality–related superiority of any floral material; such implications require targeted quantitative and biological validation.

From a comparative perspective, the present results indicate that DH-3 exhibits pronounced metabolic and transcriptional differences relative to the other materials, particularly in comparisons involving phenylpropanoid–associated features and candidate genes. Such molecular variation is useful for characterizing metabolic diversity among plant materials [39,40]; however, greater molecular divergence should not be interpreted as evidence of superior medicinal quality. The untargeted metabolomic data obtained in this study represent relative signal intensities rather than absolute compound concentrations, and gene–metabolite correlations indicate statistical associations rather than direct biochemical regulation [41]. Accordingly, the present study does not establish differences among the materials in pharmacological efficacy, bioavailability, or safety. The metabolic features and candidate genes identified here should therefore be regarded as candidates for subsequent targeted and functional validation [42], rather than as markers of material superiority.

Several limitations should be considered when interpreting these results. The study was conducted at a single location and flowering season, and the gene–metabolite integration was primarily correlation–based. In addition, the candidate genes and putatively annotated metabolic features were not independently validated by qRT–PCR or targeted quantitative LC–MS/MS. Future validation using independent samples will therefore be important to confirm the robustness and biological relevance of these associations.

## 5. Conclusions

In conclusion, integrated metabolomic and transcriptomic analyses revealed differences in untargeted metabolic features and gene–expression profiles among the three *D. huoshanense* germplasm materials, with the most pronounced differences observed between DH-2 and DH-3. Phenylpropanoid biosynthesis was recurrently enriched in the DEG comparisons, and several putative pathway–related genes, including *PAL*, *C4H*, *4CL/4CL–like*, *HCT–like*, *CSE–like*, *COMT/OMT–like*, and *CAD*, were associated with variation in phenylpropanoid–related metabolic features. Because these findings are based on relative untargeted metabolomic data and correlation analyses, they should be regarded as exploratory and do not establish direct biochemical regulation, absolute compound abundance, or differences in medicinal quality. Nevertheless, the resulting dataset provides a basis for further validation of selected genes and metabolic features through targeted quantification, functional assays, and independent sampling.

## Data Availability

The raw sequence data reported in this paper have been deposited in the Genome Sequence Archive (Genomics, Proteomics & Bioinformatics 2025) in National Genomics Data Center (Nucleic Acids Res 2025), China National Center for Bioinformation/Beijing Institute of Genomics, Chinese Academy of Sciences (GSA: CRA041969) that are publicly accessible at https://ngdc.cncb.ac.cn/gsa (accessed on 2 August 2026).

## Supplementary Materials

The following supporting information can be downloaded at: https://www.mdpi.com/article/10.3390/metabo16090612/s1, Figure S1. Permutation validation of the OPLS–DA models for pairwise comparisons among the three *D. huoshanense* germplasm materials. (A) DH-1 vs. DH-2; (B) DH-1 vs. DH-3; and (C) DH-2 vs. DH-3. Red circles and blue triangles represent R^2^Y(cum) and Q^2^(cum), respectively, and dashed lines indicate the corresponding regression trends; Figure S2: Number of up– and downregulated DEGs in three pairwise comparisons; Figure S3: GO enrichment analysis of differentially expressed genes (DEGs); Table S1: List of putatively annotated metabolic features detected in flowers of the three *D. huoshanense* germplasm materials; Table S2: Top 100 metabolic features contributing to PCA in flowers of the three *D. huoshanense* germplasm materials; Table S3: Summary statistics of the OPLS–DA models for pairwise comparisons among the three *D. huoshanense* germplasm materials; Table S4: List of DAMs in three pairwise comparisons; Table S5: Venn analysis of shared and specific DAMs among the three pairwise comparisons; Table S6: UpSet analysis of features specifically more highly and less accumulated among the three materials; Table S7: Transcriptome sequencing data summary for flower samples of three *D. huoshanense* germplasm materials; Table S8: Summary of DEGs in the three pairwise comparisons; Table S9: Top 30 GO enrichment analysis results of DEGs in the three pairwise comparisons; Table S10: Correlations between phenylpropanoid biosynthesis–related DEGs and putatively annotated phenylpropanoid– and polyketide–related metabolic features in the three pairwise comparisons.

## References

[1] Gao L., Wang F., Hou T., Geng C., Xu T., Han B., Liu D. (2022). *Dendrobium huoshanense* C.Z. Tang et S.J. Cheng: A review of its traditional uses, phytochemistry, and pharmacology. Front. Pharmacol..

[2] Zhao Y., Han B., Peng H., Wang X., Chu S., Dai J., Peng D. (2017). Identification of “Huoshan Shihu” Fengdou: Comparative Authentication of the Daodi Herb *Dendrobium huoshanense* and its Related Species by Macroscopic and Microscopic Features. Microsc. Res. Tech..

[3] Ma R., Huang Z., Zhang Z., Lu R., Li M., Luo Z., Li M., Zhang P., Lin X., Zhang G. (2025). Traditional uses, polysaccharide pharmacology, and active components biosynthesis regulation of *Dendrobium officinale*: A review. Phyton.

[4] Zha X., Luo J., Jiang S. (2007). Induction of immunomodulating cytokines by polysaccharides from *Dendrobium huoshanense*. Pharm. Biol..

[5] Yuan Y., Yu M., Jia Z., Song X., Liang Y., Zhang J. (2018). Analysis of *Dendrobium huoshanense* transcriptome unveils putative genes associated with active ingredients synthesis. BMC Genom..

[6] Li Q., Jiang H., Li Q., Zha X., Wu D., Pan L., Duan J., Liu J., Luo J. (2020). Anti-inflammatory bibenzyls from the stems of *Dendrobium huoshanense* via bioassay guided isolation. Nat. Prod. Res..

[7] Liu L., Han R., Yu N., Zhang W., Xing L., Xie D., Peng D. (2018). A method for extracting high-quality total RNA from plant rich in polysaccharides and polyphenols using *Dendrobium huoshanense*. PLoS ONE.

[8] Chen S., Dai J., Jiang X., Song X., Chen C., Chen N., Guo L., Han B. (2019). Diversity and difference of endophytes in *Dendrobium huoshanense* with different growth years. Chin. J. Chin. Mat. Med..

[9] Wang Y., Dai J., Chen R., Song C., Wei P., Wang Y., Cai Y., Han B. (2021). Long noncoding RNA-based drought regulation in the important medicinal plant *Dendrobium huoshanense*. Acta Physiol. Plant..

[10] Hao K., Wang Q., Wei S., Si H., Hao J., Chen N., Chen N., Gao X., Liao S., Zheng S. (2025). Structural characterization and anti-inflammatory activity of a neutral polysaccharide from *Dendrobium huoshanense* CZ Tang et SJ Cheng. Int. J. Biol. Macromol..

[11] Li M., Trapika I.G.S.C., Tang S.Y.S., Cho J.L., Qi Y., Li C., Li Y., Yao M., Yang D., Liu B. (2022). Mechanisms and active compounds polysaccharides and bibenzyls of medicinal *Dendrobiums* for diabetes management. Front. Nutr..

[12] Zhou P., Pu T., Gui C., Zhang X., Gong L. (2020). Transcriptome analysis reveals biosynthesis of important bioactive constituents and mechanism of stem formation of *Dendrobium huoshanense*. Sci. Rep..

[13] Yuan Y., Zuo J., Zhang H., Li R., Yu M., Liu S. (2022). Integration of transcriptome and metabolome provides new insights to flavonoids biosynthesis in *Dendrobium huoshanense*. Front. Plant Sci..

[14] Chen L., Duan S., Huang J., Hu L., Liu S., Lan Q., Wei G. (2025). Integrated metabolomic and transcriptomic analysis reveals variation in the metabolites of *Dendrobium officinale*, *Dendrobium huoshanense*, *Dendrobium nobile*. Phytochem. Anal..

[15] Zhao M., Fan J., Liu Q., Luo H., Tang Q., Li C., Zhao J., Zhang X. (2021). Phytochemical profiles of edible flowers of medicinal plants of *Dendrobium officinale* and *Dendrobium devonianum*. Food Sci. Nutr..

[16] Li Q., Yang X., Zha X., Pan L., Zang D., Zhang F., Luo J. (2022). Protective effects of three flavonoids from *Dendrobium huoshanense* flowers on alcohol-induced hepatocyte injury via activating Nrf2 and inhibiting NF-κB pathways. Chem. Biodivers..

[17] Ren X., Huang Y., Zhang L., Lu Q., Wang X., Ying W., Ruan S. (2025). Integrated analysis of transcriptomics and metabolomics of *Dendrobium officinale* flowers at different developmental stages. Sci. Rep..

[18] He C., Liu X., Teixeira da Silva J.A., Liu N., Zhang M., Duan J. (2020). Transcriptome sequencing and metabolite profiling analyses provide comprehensive insight into molecular mechanisms of flower development in *Dendrobium officinale* (Orchidaceae). Plant Mol. Biol..

[19] Hu L., Wang S., Zhang L., Shang L., Zong R., Li J., Wu Z., Meng Y., Dai Y., Huang Y. (2024). Wild imitating vs greenhouse cultivated *Dendrobium huoshanense*: Chemical quality differences. PLoS ONE.

[20] Liu S., Zhang H., Yuan Y. (2022). A comparison of the flavonoid biosynthesis mechanisms of *Dendrobium* species by analyzing the transcriptome and metabolome. Int. J. Mol. Sci..

[21] Yuan Y., Zuo J., Wan X., Zhou R., Xing W., Liu S. (2024). Multi-omics profiling reveal responses of three major *Dendrobium* species from different growth years to medicinal components. Front. Plant Sci..

[22] Yuan Y., Zuo J., Zhang H., Zu M., Yu M., Li S. (2022). Transcriptome and metabolome profiling unveil the accumulation of flavonoids in *Dendrobium officinale*. Genomics.

[23] Tao S., Wang H., Ji Q., Yang Y., Wei G., Li R., Zhou B. (2025). Integration of metabolomics and transcriptomics to reveal the antitumor mechanism of *Dendrobium officinale* polysaccharide-based nanocarriers in enhancing photodynamic immunotherapy in colorectal cancer. Pharmaceutics.

[24] Zhan X., Qi J., Zhou B., Mao B. (2020). Metabolomic and transcriptomic analyses reveal the regulation of pigmentation in the purple variety of *Dendrobium officinale*. Sci. Rep..

[25] Brunius C., Shi L., Landberg R. (2016). Large-scale untargeted LC-MS metabolomics data correction using between-batch feature alignment and cluster-based within-batch signal intensity drift correction. Metabolomics.

[26] Yao T., Feng K., Xie M., Barros J., Tschaplinski T.J., Tuskan G.A., Muchero W., Chen J. (2021). Phylogenetic occurrence of the phenylpropanoid pathway and lignin biosynthesis in plants. Front. Plant Sci..

[27] Ninkuu V., Aluko O.O., Yan J., Zeng H., Liu G., Zhao J., Li H., Chen S., Dakora F.D. (2025). Phenylpropanoids metabolism: Recent insight into stress tolerance and plant development cues. Front. Plant Sci..

[28] Vogt T. (2010). Phenylpropanoid biosynthesis. Mol. Plant.

[29] Liu Q., Luo L., Zheng L. (2018). Lignins: Biosynthesis and biological functions in plants. Int. J. Mol. Sci..

[30] Fraser C.M., Chapple C. (2011). The phenylpropanoid pathway in Arabidopsis. Arab. Book.

[31] Hoffmann L., Besseau S., Geoffroy P., Ritzenthaler C., Meyer D., Lapierre C., Pollet B., Legrand M. (2004). Silencing of hydroxycinnamoyl-coenzyme A shikimate/quinate hydroxycinnamoyltransferase affects phenylpropanoid biosynthesis. Plant Cell.

[32] Vanholme R., Cesarino I., Rataj K., Xiao Y., Sundin L., Goeminne G., Kim H., Cross J., Morreel K., Araujo P. (2013). *Caffeoyl shikimate esterase* (CSE) is an enzyme in the lignin biosynthetic pathway in Arabidopsis. Science.

[33] Boerjan W., Ralph J., Baucher M. (2003). Lignin biosynthesis. Annu. Rev. Plant Biol..

[34] Tohge T., Watanabe M., Hoefgen R., Fernie A.R. (2013). The evolution of phenylpropanoid metabolism in the green lineage. Crit. Rev. Biochem. Mol. Biol..

[35] Zhang Y., Butelli E., Martin C. (2014). Engineering anthocyanin biosynthesis in plants. Curr. Opin. Plant Biol..

[36] Yan L., Wang X., Liu H., Tian Y., Lian J., Yang R., Hao S., Wang X., Yang S., Li Q. (2015). The genome of *Dendrobium officinale* illuminates the biology of the important traditional Chinese orchid herb. Mol. Plant.

[37] Zha X., Luo J., Luo S., Jiang S. (2007). Structure identification of a new immunostimulating polysaccharide from the stems of *Dendrobium huoshanense*. Carbohydr. Polym..

[38] Ng T.B., Liu J., Wong J.H., Ye X., Sze S.C.W., Tong Y., Zhang K. (2012). Review of research on *Dendrobium*, a prized folk medicine. Appl. Microbiol. Biotechnol..

[39] Rai A., Saito K., Yamazaki M. (2017). Integrated omics analysis of specialized metabolism in medicinal plants. Plant J..

[40] Fang C., Fernie A.R., Luo J. (2019). Exploring the diversity of plant metabolism. Trends Plant Sci..

[41] Fernie A.R., Tohge T. (2017). The genetics of plant metabolism. Annu. Rev. Genet.

[42] Perez De Souza L., Alseekh S., Brotman Y., Fernie A.R. (2020). Network-based strategies in metabolomics data analysis and interpretation: From molecular networking to biological interpretation. Expert Rev. Proteom..

