# Supplementary material for: Integrated Metabolomic and Transcriptomic Analyses Reveal Phenylpropanoid–Associated Variation Among Three Dendrobium huoshanense Germplasm Materials
Source: Metabolites. 2026 Aug 27;16(9):612. doi: 10.3390/metabo16090612 (PMC13609788; doi:10.3390/metabo16090612)
Supplement: Supplementary file 1 [file metabolites-16-00612-s001.zip › Figure S1.pdf]

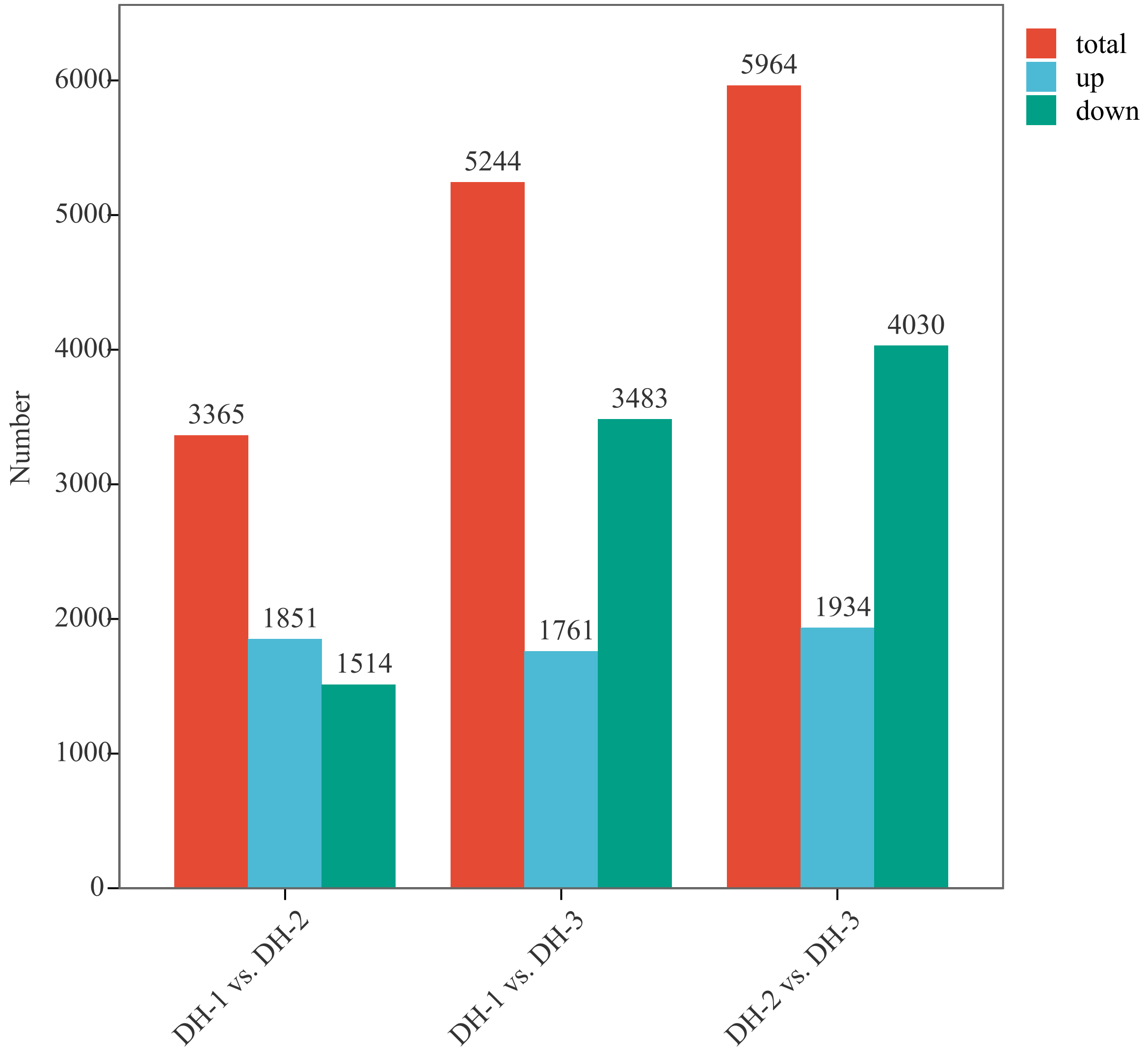

Figure S1. Number of up- and downregulated DEGs in three pairwise comparisons.
