# Supplementary material for: Integrated Metabolomic and Transcriptomic Analyses Reveal Phenylpropanoid–Associated Variation Among Three Dendrobium huoshanense Germplasm Materials
Source: Metabolites. 2026 Aug 27;16(9):612. doi: 10.3390/metabo16090612 (PMC13609788; doi:10.3390/metabo16090612)
Supplement: Supplementary file 1 [file metabolites-16-00612-s001.zip › Figure S2.pdf]

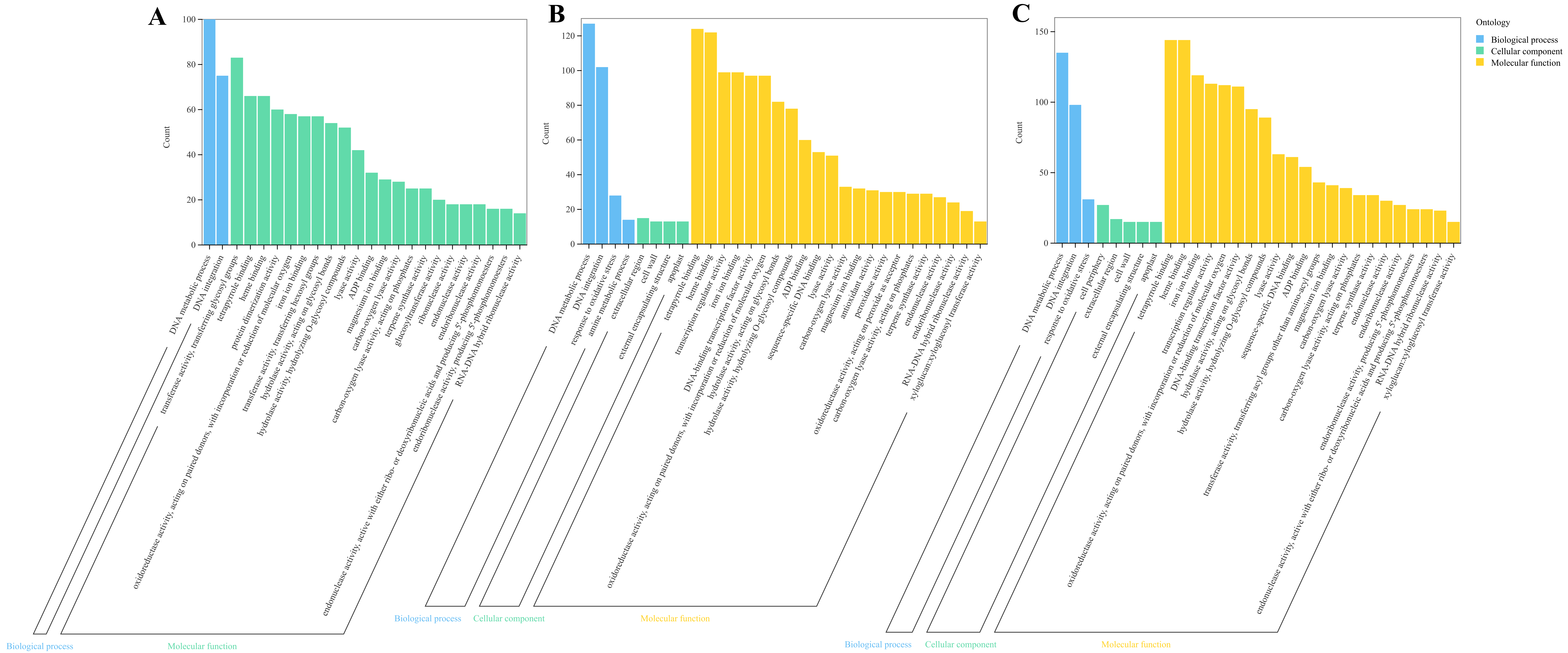

Figure S2. GO enrichment analysis of differentially expressed genes (DEGs).
The top 30 enriched GO terms are displayed for three comparison groups: (A) DH-1 vs. DH-2, (B) DH-1 vs. DH-3, and (C) DH-2 vs. DH-3. The Y-axis (Count) represents the number of DEGs associated with each term, and the X-axis lists the GO terms. The terms are categorized into three main ontologies: Biological Process , Cellular Component, and Molecular Function.
