# Supplementary material for: Integrated Metabolomic and Transcriptomic Analyses Reveal Phenylpropanoid–Associated Variation Among Three Dendrobium huoshanense Germplasm Materials
Source: Metabolites. 2026 Aug 27;16(9):612. doi: 10.3390/metabo16090612 (PMC13609788; doi:10.3390/metabo16090612)
Supplement: Supplementary file 1 [file metabolites-16-00612-s001.zip › Figure S3.pdf]

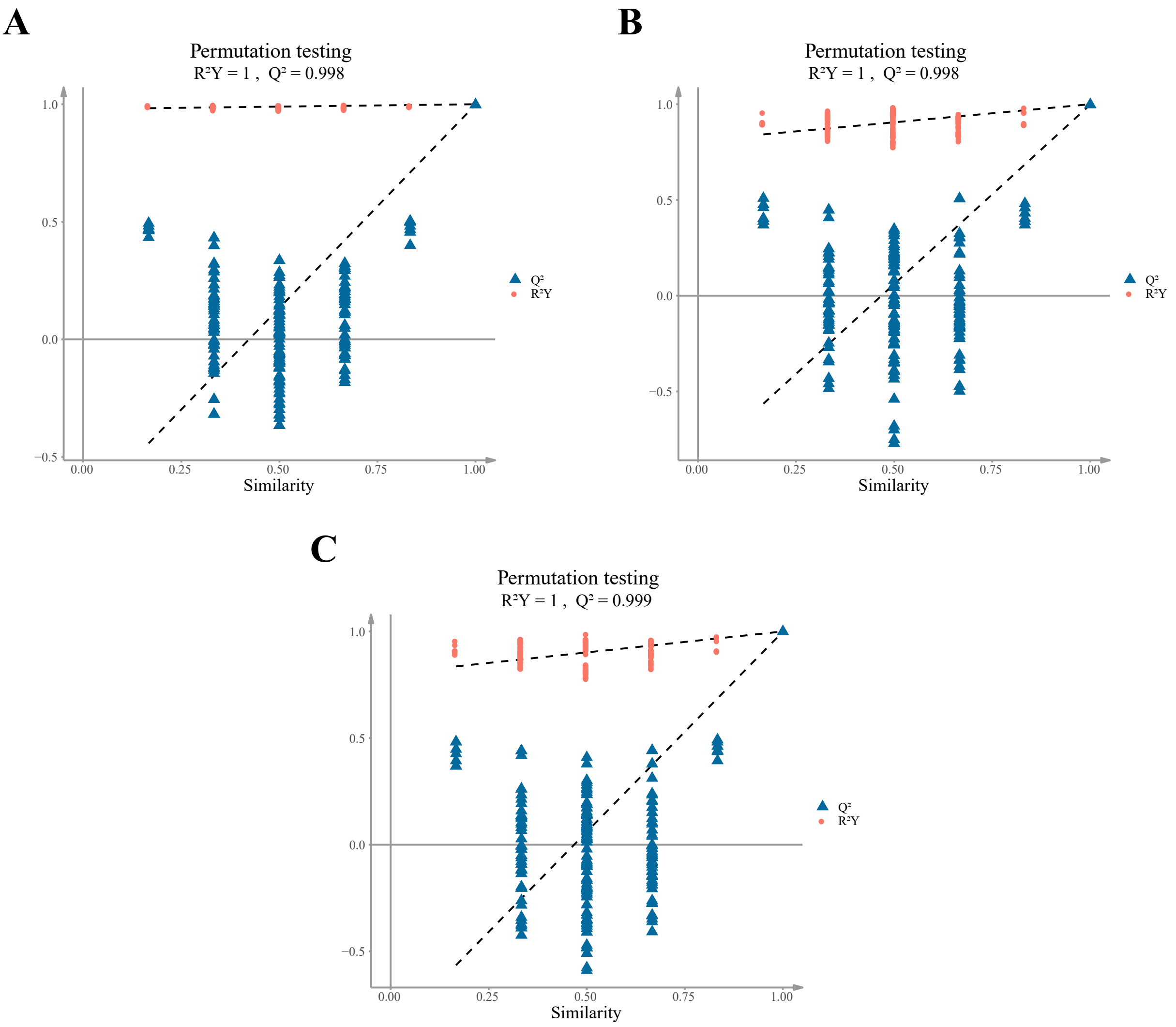

Figure S3. Permutation validation of the OPLS-DA models for pairwise comparisons among the three *D. huoshanense* materials. (A) DH-1 vs. DH-2; (B) DH-1 vs. DH-3; and (C) DH-2 vs. DH-3. Red circles and blue triangles represent R^2Y(cum) and Q^2(cum), respectively, and dashed lines indicate the corresponding regression trends.
